# Venetoclax and Hypomethylating Agents as First-line Treatment in Newly Diagnosed Patients with AML in a Predominately Community Setting in the US

**DOI:** 10.1093/oncolo/oyac135

**Published:** 2022-08-04

**Authors:** Pankit Vachhani, Evelyn M Flahavan, Tao Xu, Esprit Ma, Melissa Montez, Anda Gershon, Maika Onishi, Huan Jin, Grace Ku, Brannon Flores, Cat N Bui, Jonathan A Abbas, William Donnellan

**Affiliations:** Department of Medicine, University of Alabama at Birmingham, Birmingham, AL, USA; Roche Products Ltd., Welwyn Garden City, UK; F. Hoffmann-La Roche Ltd, Basel, Switzerland; Genentech, Inc., South San Francisco, CA, USA; Genentech, Inc., South San Francisco, CA, USA; Genentech, Inc., South San Francisco, CA, USA; Genentech, Inc., South San Francisco, CA, USA; Genentech, Inc., South San Francisco, CA, USA; Genentech, Inc., South San Francisco, CA, USA; Genentech, Inc., South San Francisco, CA, USA; AbbVie, Inc., North Chicago, IL, USA; Tennessee Oncology, Nashville, TN, USA; Sarah Cannon Research Institute, Tennessee Oncology, Nashville, TN, USA

**Keywords:** leukemia, myeloid, acute, venetoclax, bone marrow, retrospective studies, United States, duration of therapy

## Abstract

**Background:**

Treatment with venetoclax + hypomethylating agents (HMAs) is standard-of-care for newly diagnosed (ND) patients with acute myeloid leukemia (AML) aged ≥75 years, or with comorbidities precluding intensive chemotherapy. We describe real-world venetoclax + HMA treatment practices and outcomes in patients with ND AML in the US.

**Patients and Methods:**

This retrospective cohort study used an electronic health record-derived, US nationwide, de-identified database, and included adults with ND AML, initiating venetoclax + HMA treatment ≤30 days from diagnosis (June 1, 2018-January 31, 2020). Venetoclax treatment variables included dosing information, schedule modifications, and drug–drug interactions. The median venetoclax + HMA treatment duration and overall survival (OS) from venetoclax initiation to discontinuation, death, or end of follow-up (August 31, 2020) were examined by Kaplan-Meier analyses.

**Results:**

Overall, 169 patients were included. The median age at diagnosis was 77 years; 85.2% of patients were treated in community practice. Ninety-five of 169 patients (56.2%) had evaluable bone marrow response data following the start of treatment; 53.7% were assessed approximately at the end of cycle 1. Following the first treatment cycle, treatment schedule modifications were recorded in 101 patients and dose changes in 56, primarily due to toxicity. The median treatment duration was 5.2 months; the median OS was 8.6 months (median follow-up was 7.2 months). Venetoclax dose changes did not modify efficacy outcomes, but longer median OS was associated with venetoclax treatment schedule modifications (*P* = .02).

**Conclusions:**

This study reflects early real-world experience with venetoclax + HMAs in a predominantly community setting and emphasizes the importance of appropriate venetoclax management in optimizing patient outcomes.

## Teaser

Optimal dose scheduling of venetoclax may improve clinical outcomes by maintaining efficacy while reducing the frequency and severity of adverse events. This article examines the early real-world utilization of venetoclax plus hypomethylating agents for the treatment of newly diagnosed acute myeloid leukemia.

**Implications for Practice**

This study represents early real-world experience with venetoclax plus hypomethylating agents (HMAs) for newly diagnosed patients with acute myeloid leukemia (AML) in a predominantly community setting in the United States. Most patients had dose changes or modifications to their venetoclax regimen, mainly due to therapy-related toxicities. Venetoclax schedule changes and dose cycle modifications were associated with longer treatment duration and prolonged overall survival. These findings suggest that patients with AML benefit from appropriate venetoclax management (including early bone marrow assessment and treatment schedule modifications for toxicities) during treatment with venetoclax plus HMAs, to optimize patient outcomes.

## Introduction

Acute myeloid leukemia (AML) is an aggressive myeloid neoplasm, primarily affecting the elderly,^1,2^ with a median age at the time of diagnosis of 68 years.^3^ An estimated 20 240 new cases of AML were expected to occur in the US in 2021.^3^ Historically, patients with AML who are older or ineligible for intensive induction chemotherapy had limited treatment options and poor survival outcomes.^4-7^

Combination therapy for AML with the venetoclax + hypomethylating agents (HMAs; azacitidine or decitabine) has been approved in the US since 2018 (accelerated approval by the US Food and Drug Administration in November 2018, with subsequent full approval in October 2020).^8-12^ In the phase Ib study (NCT02203773) of venetoclax + HMAs in patients aged ≥65 years with treatment-naïve AML ineligible for intensive induction chemotherapy, an overall response rate of 68.3% was achieved (including complete response [CR], CR with incomplete hematologic recovery [CRi] and partial response across all evaluated doses). The median response duration was 11.3 months, and the median overall survival (OS) was 17.5 months (median follow-up 15.1 months).^13^ Similarly, the phase III VIALE-A trial showed that first-line (1L) treatment with venetoclax + azacitidine (versus placebo + azacitidine) in older (≥75 years of age) or unfit patients with newly diagnosed AML ineligible for intensive induction chemotherapy improved median OS (14.7 months versus 9.6 months, respectively; hazard ratio for death, 0.66; *P* < .001) and response rates (66.4% versus 28.3%, respectively; *P* < .001).^11^ Following the efficacy demonstrated in clinical trials, treatment with venetoclax + HMAs is now considered the standard-of-care for patients with newly diagnosed AML who are older or have comorbidities precluding intensive induction chemotherapy.^12^

Hematologic adverse events (all grades) occurred in 83% of patients in the venetoclax + azacitidine arm of VIALE-A.^11^ Cytopenias, or reduction in the number of blood cells, are common in AML and are associated both with the disease nature^14,15^ and treatment with venetoclax and/or HMAs.^11,16-20^ Data from VIALE-A showed that the majority of patients with CR/CR with partial hematologic recovery (CRh) required venetoclax dosing (schedule) modifications to manage cytopenias; these were most commonly cycle delays or reductions in dosing duration (eg, 21 days of a 28-day dosing schedule).^21^ Insights from early adoption of venetoclax + HMAs in clinical practice show that frequent dose interruptions and conversion from 28-day dosing to shorter venetoclax dosing schedules (eg, 21-day or 14-day schedules) have been implemented to manage cytopenias.^22,23^ In addition, dose modifications and administration of granulocyte colony-stimulating factor can be adopted as preventative measures to mitigate the risk of cytopenias.^23^ Further, dose reductions are recommended for patients receiving venetoclax with cytochrome P450 inhibitors, for moderate drug–drug interactions.^24^

Optimal dose scheduling of venetoclax may improve clinical outcomes by maintaining efficacy while reducing the frequency and severity of cytopenias. Here we examine the early real-world utilization of venetoclax + HMAs for the treatment of newly diagnosed AML in a predominantly community setting in the US. Treatment patterns assessed included venetoclax total treatment duration and modifications of venetoclax daily dose and treatment schedule. The documented reasons for venetoclax daily dose and treatment schedule modifications, including discontinuation, were investigated. This analysis also describes the real-world management of cytopenias and examines patient outcomes.

## Materials and Methods

### Data Source

This retrospective cohort study used data from the Flatiron Health database: the US nationwide, electronic health record (EHR)-derived longitudinal database, which is comprised of de-identified, patient-level structured and unstructured data, curated via technology-enabled abstraction. During the study period, the de-identified data originated from approximately 280 US cancer clinics (~800 sites of care).^25,26^ The database for AML was developed specifically to include unstructured data needed to describe the AML patient trajectory, including baseline characteristics, prior medical history, treatment details, and molecular and cytogenetic markers for risk stratification.

### Patient Cohort

The cohort for the current analysis was selected from the Flatiron Health AML database, with additional chart review and review of physical notes to better understand venetoclax treatment patterns. As of April 2020, this database included over 3300 patients diagnosed with AML (in line with the International Classification of Disease V.9, see Supplementary Methods), with at least 2 documented clinical visits for AML (academic or community encounters on different days) in the Flatiron Health network since January 1, 2014.^27^

Patients were included in the current analysis if they were ≥18 years old with newly diagnosed AML and had received 1L treatment with venetoclax + azacitidine or venetoclax + decitabine (± temporary use of hydroxyurea for white blood cell reduction) in routine clinical practice within 30 days of the AML diagnosis, any time between June 1, 2018 and January 31, 2020 (with follow-up until August 31, 2020). Patients with missing unstructured documents (ie, documents with no predefined format or organization, such as pathology) that precluded a confirmation of the AML diagnosis were excluded from the analyses. Also excluded were patients diagnosed with acute promyelocytic leukemia or with a positive *t*(9;22) cytogenetic test on or before the start of 1L therapy, or patients with any prior venetoclax exposure.

Five patient cohorts of interest were considered: the all-comers cohort (patients with newly diagnosed AML treated with 1L venetoclax + HMAs) and 4 sub-cohorts: (i) bone marrow (BM) blast clearance (patients with ≤5% blasts in the BM during follow-up [between June 2018 and August 2020]), (ii) real-world CR/CRh (rwCR/CRh; patients from the BM blast clearance [≤5% blasts] sub-cohort with least partial count recovery [platelet count >50 × 10^9^/L and absolute neutrophil count >0.5 × 10^9^/L within ± 14 days of the blast assessment] during follow-up), and (iii) non-response (patients with >5% blasts in the BM during follow-up), and (iv) patients who did not have evaluable blast clearance (no BM data found in their records during follow-up). Sub-cohorts i-iii comprised the BM blast-evaluable group.

Institutional Review Board approval of the study protocol was obtained from WIRB-Copernicus Group prior to study conduct and included a waiver of informed consent.

### Variables

Patient demographics, baseline characteristics, disease-related information, and treatments were captured as per the Flatiron Health AML database. The European LeukemiaNet (ELN) 2017 risk classification^4^ was derived from available cytogenetic and molecular data in the database. Additional variables related to venetoclax treatment were manually abstracted from EHR unstructured data, including dosing information, schedule modifications, and drug–drug interactions, with the dose on day 7 taken as the target starting dose due to the initial dose ramp-up (over 3 days) for tumor lysis syndrome monitoring (to allow for any deviation/delay of ramp-up from label guidance). Dosing cycles were derived for a patient’s treatment period based on the first administration of HMAs (cycle 1, day 1) and on a cadence of 28 days (±3 days). These variables and other information collated from the structured and abstracted treatment data are available in Supplementary Table S1. We examined venetoclax treatment modifications, grouped as dose (level) changes and treatment schedule changes (in-cycle interruptions, cycle delays, and dose schedule per cycle changes).

### Statistical Analysis

Analyses of patient and treatment (venetoclax + HMA) characteristics were descriptive for all cohorts examined, and focused on differences within the venetoclax-treated patient population. To eliminate the potential to re-identify a patient, any instances where there are fewer than 4 patients for a particular characteristic or variable have been described as such (as per the threshold accepted by the National Center for Health Statistics and the Agency for Healthcare Research and Quality, detailed in the Federal Committee’s Statistical Policy, 2005). Further, the date of death is recorded as month/year to protect patient privacy and prevent potential re-identification of patients. Reasons for dose changes and treatment schedule modifications were examined where reported. Dose schedule modifications were examined relative to the timing of treatment response (CR/CRh) and the occurrence of treatment-emergent cytopenia (in patients without cytopenias at baseline).

For the all-comer and sub-cohorts of interest, the Kaplan-Meier method was used to determine median OS (with 95% confidence intervals calculated using the Brookmeyer–Crowley method), from the date of 1L venetoclax + HMA treatment administration to the month of death (censoring occurred at last visit date or last treatment administration date on or before end of follow-up). Log-rank tests were conducted to detect potential differences between the sub-cohorts of interest, and Kaplan-Meier analyses were used to describe the median duration of venetoclax treatment (from the date of 1L treatment initiation to the end of treatment). Within the all-comers cohort, outcomes were evaluated according to whether patients had dose changes (eg, venetoclax change from 400 mg to 200 mg) or treatment schedule modifications (eg, change in dose from 28 to 21 days out of a 28-day cycle). Additional subgroup outcome analyses were conducted by age (<75 versus ≥75 years), practice type (academic versus community), and type of AML (secondary AML [s-AML] versus de-novo) in the all-comers cohort, as well as in patients with s-AML according to the presence or absence of prior treatment with HMAs.

Cox regression analyses with time-varying covariates (time of first treatment schedule or dose modification) were conducted to examine the association between dose changes and treatment schedule modifications, and the duration of treatment and OS outcomes. A *P*-value < .05 is considered statistically significant.

## Results

### Baseline Characteristics

**Figure 1.**
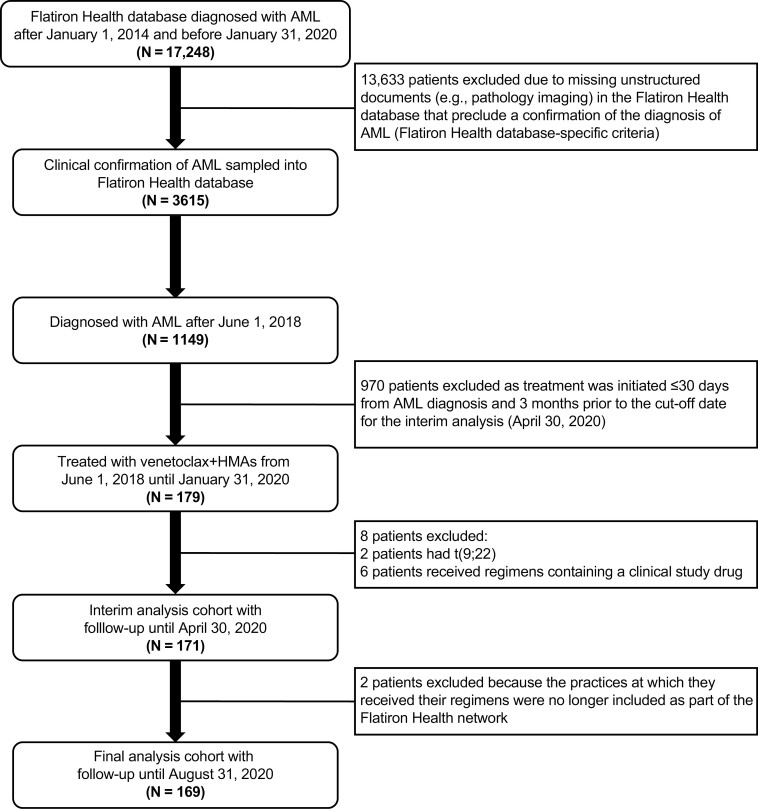
Attrition of cohort from the Flatiron Health network.5. Abbreviations: AML, acute myeloid leukemia; HMAs, hypomethylating agents.

**Figure 2.**
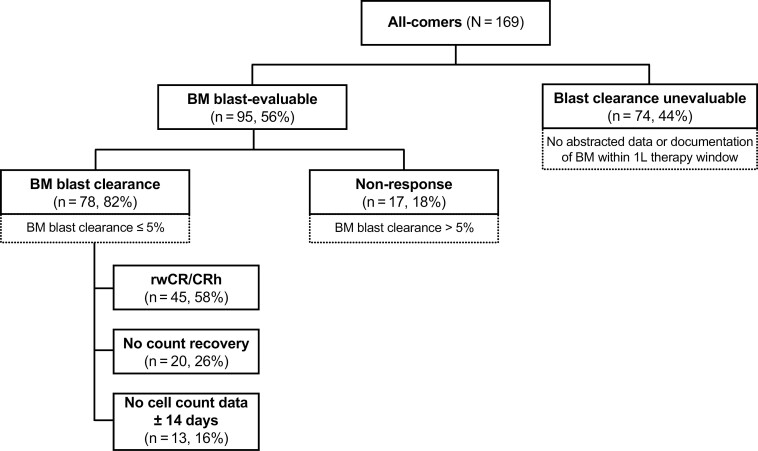
Stratification into the pre-specified cohorts. Blast clearance is defined as ≤5% blasts in the BM after the start of 1L therapy and before the end of 1L therapy. rwCR/CRh is defined as ≤5% blasts in the BM with at minimum partial count recovery (platelet count >50 × 10^9^/L and absolute neutrophil count >0.5 × 10^9^/L) within the period ± 14 days of the blast assessment. Abbreviations: 1L, first-line; BM, bone marrow; rwCR/CRh, real-world complete response/complete response with partial hematologic recovery.

A total of 169 patients were included in the all-comers cohort (Fig. 1) and subsequently stratified into the pre-specified sub-cohorts (Fig. 2). Balanced demographic and baseline characteristics were observed across the cohorts (Table 1).

**Table 1.** Patient characteristics at baseline

	All-comers (*N* = 169)	BM blast-evaluable (*n* = 95; 56%)			Blast clearance nevaluable^b^ (*n* = 74; 44%)
		BM blast clearance (*n* = 78)	rwCR/CRh (*n* = 45)^a^	Non-response (*n* = 17)	
Age at diagnosis, years					
Median (IQR)	77.0 (72.0-82.0)	77.0 (71.3-80.0)	77.0 (70.0-80.0)	75.0 (71.0-80.0)	77.5 (73.0-83.8)
Min, max	39.0, 85.0	53.0, 85.0	53.0, 85.0	39.0, 84.0	58.0, 85.0
Age at diagnosis (categories), years					
18-64	14 (8.3)	6 (7.7)	5 (11.1)	<4 (<23.5)	6 (8.1)
65-74	49 (29.0)	24 (30.8)	13 (28.9)	6 (35.3)	19 (25.7)
≥75	106 (62.7)	48 (61.5)	27 (60.0)	9 (52.9)	49 (66.2)
Sex					
Female	74 (43.8)	38 (48.7)	21 (46.7)	8 (47.1)	28 (37.8)
Male	95 (56.2)	40 (51.3)	24 (53.3)	9 (52.9)	46 (62.2)
Year of diagnosis					
2018	31 (18.3)	17 (21.8)	11 (24.4)	4 (23.5)	10 (13.5)
2019	128 (75.7)	58 (74.4)	33 (73.3)	13 (76.5)	57 (77.0)
2020	10 (5.9)	<4 (<5.1)	<4 (<8.9)	0 (0)	7 (9.5)
AML type					
De-novo AML	95 (56.2)	44 (56.4)	27 (60.0)	11 (64.7)	40 (54.1)
s-AML	74 (43.8)	34 (43.6)	18 (40.0)	6 (35.3)	34 (45.9)
MDS	59 (34.9)	26 (33.3)	15 (33.3)	6 (35.3)	27 (36.5)
CMML	8 (4.7)	5 (6.4)	<4 (<8.9)	0 (0)	<4 (<5.4)
Other^c^	7 (4.1)	<4 (<5.1)	<4 (<8.9)	0 (0)	<4 (<5.4)
Therapy-related AML^d^	14 (8.3)	7 (9.0)	4 (8.9)	4 (23.5)	<4 (<5.4)
Practice type					
Academic	25 (14.8)	16 (20.5)	9 (20.0)	<4 (<23.5)	6 (8.1)
Community	144 (85.2)	62 (79.5)	36 (80.0)	14 (82.4)	68 (91.9)
Prior malignancy					
Yes	52 (30.8)	23 (29.5)	14 (31.1)	11 (64.7)	18 (24.3)
No	117 (69.2)	55 (70.5)	31 (68.9)	6 (35.3)	56 (75.7)
Blast assessment at diagnosis					
Yes	160 (94.7)	73 (93.6)	43 (95.6)	16 (94.1)	71 (95.9)
No	9 (5.3)	5 (6.4)	<4 (<8.9)	<4 (<23.5)	<4 (<5.4)
BM blast %					
≤30	66 (39.1)	27 (34.6)	12 (26.7)	7 (41.2)	32 (43.2)
31-50	40 (23.7)	22 (28.2)	14 (31.1)	5 (29.4)	13 (17.6)
>50	54 (32.0)	24 (30.8)	17 (37.8)	4 (23.5)	26 (35.1)
Unknown/undocumented	9 (5.3)	5 (6.4)	<4 (<8.9)	<4 (<23.5)	<4 (<5.4)
Tissue for diagnosis					
BM	148 (87.6)	71 (91.0)	41 (91.1)	14 (82.4)	63 (85.1)
PB	18 (10.7)	7 (9.0)	4 (8.9)	3 (17.6)	8 (10.8)
Unknown/undocumented	<4 (<2.4)	0 (0)	0 (0)	0 (0)	<4 (<5.4)
ECOG PS					
0	32 (18.9)	15 (19.2)	6 (13.3)	4 (23.5)	13 (17.6)
1	68 (40.2)	33 (42.3)	21 (46.7)	6 (35.3)	29 (39.2)
2	33 (19.5)	14 (17.9)	10 (22.2)	<4 (<23.5)	16 (21.6)
3+	13 (7.7)	5 (6.4)	<4 (<8.9)	<4 (<23.5)	7 (9.5)
Missing	23 (13.6)	11 (14.1)	5 (11.1)	<4 (<23.5)	9 (12.2)
ELN 2017 classification					
Favorable	22 (13.0)	13 (16.7)	8 (17.8)	<4 (<23.5)	7 (9.5)
Intermediate	37 (21.9)	18 (23.1)	11 (24.4)	<4 (<23.5)	16 (21.6)
Adverse	65 (38.5)	29 (37.2)	14 (31.1)	11 (64.7)	25 (33.8)
Inconclusive	45 (26.6)	18 (23.1)	12 (26.7)	<4 (<23.5)	26 (35.1)

All values are *n* (%) unless otherwise specified. To eliminate the potential to re-identify a patient, any instances where there are fewer than 4 patients have been defined as < 4.

rwCR/CRh is a subset of the 78 patients with BM blast clearance; among all the patients with BM clearance, 13 patients had no cell count data within the ± 14-day time frame. The remaining BM blast-evaluable patients (*n* = 17) had a non-response.

Patients who did not have evaluable blast clearance due to a lack of BM data found in their records during follow-up.

Including chronic myelogenous leukemia (*n* = 1), essential thrombocythemia (*n* = 2), myelofibrosis (*n* = 1), polycythemia vera (*n* = 1), and other prior hematologic diseases (*n* = 2).

Both de-novo AML and s-AML could be therapy-related.

Abbreviations: AML, acute myeloid leukemia; BM, bone marrow; CMML, chronic myelomonocytic leukemia; ECOG PS, Eastern Cooperative Oncology Group performance status; ELN, European LeukemiaNet; IQR, interquartile range; max, maximum; MDS, myelodysplastic syndrome; min, minimum; PB, peripheral blood; rwCR/CRh, real-world complete response/complete response with partial hematologic recovery; s-AML, secondary acute myeloid leukemia.

For the all-comers cohort, the median age at diagnosis was 77 years. At baseline, 27.2% of patients had an Eastern Cooperative Oncology Group performance status (ECOG PS) ≥2 and 43.8% had s-AML. The ELN 2017 risk classification was favorable for 13.0% of the all-comers cohort, intermediate for 21.9%, and adverse for 38.5%. Most patients (85.2%) were treated at a community (versus academic) oncology practice (Table 1).

### Treatment and Follow-up

Most patients in the all-comers cohort received venetoclax + azacitidine (103 [60.9%]), and the remainder received venetoclax + decitabine (66 [39.1%] patients; Table 2). Median follow-up was 7.2 months (interquartile range [IQR] = 3.1-10.5) for the all-comers cohort versus 4.6 months (IQR = 3.8-8.6) and 3.7 months (IQR = 2.1-7.8) in the non-response sub-cohort and the blast clearance unevaluable sub-cohort, respectively. Approximately half of the patients (94 [55.6%]) had died by the end of follow-up; 17 (10.1%) patients died within 60 days of 1L treatment initiation. Notably, the highest rates of early deaths (≤60 days of starting 1L treatment) were reported in the blast clearance unevaluable sub-cohort (18.9%; Table 2). In comparison, less than 4 (<4.2%) of the 95 patients with documented BM biopsy (BM blast-evaluable) had early mortality (≤60 days of starting 1L treatment; Table 2). Death within 30 days of treatment initiation was low, occurring in 4 or less (≤2.4%) patients in the all-comers cohort.

**Table 2.** Post-baseline treatment, follow-up, and outcomes of cohort by response groups

	All-comers (*n* = 169)	BM blast-evaluable (*n* = 95; 56%)			Blast clearance unevaluable^b^ (*n* = 74; 44%)
		BM blast clearance (*n* = 78)	rwCR/CRh (*n* = 45)^a^	Non-response (*n* = 17)	
1L regimen					
Venetoclax + azacitidine	103 (60.9)	49 (62.8)	30 (66.7)	11 (64.7)	43 (58.1)
Venetoclax + decitabine	66 (39.1)	29 (37.2)	15 (33.3)	6 (35.3)	31 (41.9)
Transplant (any time during the study period)					
Yes	12 (7.1)	11 (14.1)	7 (15.6)	0 (0)	<4 (<5.4)
Death in follow-up	94 (55.6)	29 (37.2)	12 (26.7)	14 (82.4)	51 (68.9)
Death within 60 days	17 (10.1)	<4 (<5.1)	0 (0)	<4 (<23.5)	14 (18.9)
Follow-up time^c^					
Median (IQR)	7.2 (3.1−10.5)	9.6 (6.6−14.2)	10.5 (7.9−15.6)	4.6 (3.8−8.6)	3.7 (2.1−7.8)
Min, max	0.6, 24.8	1.9, 24.8	2.2, 24.8	0.8, 11.4	0.6, 19.2

All values are *n* (%) unless otherwise specified. To eliminate the potential to re-identify a patient, any instances where there are fewer than 4 patients have been defined as <4.

rwCR/CRh is a subset of the 78 patients with BM blast clearance; among all the patients with BM clearance, 13 patients had no cell count data within the ±14-day time frame. The remaining BM blast-evaluable patients (*n* = 17) had a non-response.

Patients who did not have evaluable blast clearance due to a lack of BM data found in their records during follow-up.

Months from treatment initiation to death or censoring.

Abbreviations: 1L, first-line; BM, bone marrow; IQR, interquartile range; max, maximum; min, minimum; rwCR/CRh, real-world complete response/complete response with partial hematologic recovery.

### Venetoclax Dosing

The post-ramp-up dose of venetoclax after day 7 was 400 mg (83 [49.1%] patients), 300 mg (5 [3.0%] patients), 200 mg (33 [19.5%] patients), or ≤100 mg (34 [20.1%] patients), with 14 (8.3%) patients having no dose recorded in the EHR. Dosing intensity (the proportion of days with venetoclax dosing from the recorded start date to end date of treatment) was 67.8% (IQR = 45.3-87.0). Following venetoclax dose ramp-up, 56 patients (33.1%) had changes in venetoclax dose.

Overall, 149 (88.2%) patients started on the 28-day schedule for venetoclax (Supplementary Fig. S1); the remaining patients either started on 21-day or 14-day schedules, with the exception of 6 patients whose schedule information was unknown. Most patients (110/149 [73.8%]) did not deviate from the 28-day schedule; however, 48/110 (43.6%) patients who did not deviate from the 28-day dosing schedule for venetoclax received ≤60 days of therapy. A total of 101 (59.8%) patients had a treatment schedule modification, with a median time to first schedule modification of 33 days (95% CI = 28-52), occurring at approximately 1-2 treatment cycles. Dose cycle conversion (patients who converted to a cycle of ≤21 days venetoclax dosing for 2 consecutive cycles) was reported for 39/149 (26.2%) patients; 24 (16.1%) patients switched from 28-day to 21-day schedules of venetoclax per HMA cycle and 13 (8.7%) patients switched to 14-day venetoclax schedules per HMA cycle (at any point). Further, 89 (52.7%) patients discontinued venetoclax treatment (12 patients from the non-response subgroup [*n* = 17], 35 patients from the BM blast clearance subgroup [*n* = 78] and 42 patients in the blast clearance unevaluable subgroup [*n* = 74]).

The most commonly cited reasons for dose changes, treatment schedule modifications, and discontinuation was the toxic effect of therapy (specific adverse events were not abstracted from the EHR). Overall, 14 (25%) patients with dose changes had drug–drug interaction as a documented reason. However, the reason was not reported for 35 (62.5%) patients who had a dose change. Of the patients who discontinued therapy 11 (12.4%), patients had documented progression (Table 3). Notably, baseline characteristics of patients with dose changes or treatment schedule modifications were not significantly different from those who did not modify their venetoclax treatment (data not shown).

**Table 3.** Reasons for dose changes, dose schedule modification, and discontinuation in the all-comers cohort^a^

	Dose change (*n* = 56)	Dose schedule modification (*n* = 101)	Discontinuation (*n* = 89)
Total			
No. of events	92	236	89
Toxic effect of therapy			
No. of patients (%)	21 (37.5)	79 (78.2)	41 (46.1)
No. of events	27	173	41
Disease-related symptoms not due to therapy			
No. of patients (%)		9 (8.9)	9 (10.1)
No. of events		9	9
Drug–drug interaction			
No. of patients (%)	14 (25.0)	4 (4.0)	
No. of events	18	4	
Progression			
No. of patients (%)		<4 (<4.0)	11 (12.4)
No. of events		<4	11
No evidence of disease			
No. of patients (%)	<4 (<7.1)	5 (5.0)	<4 (<4.5)
No. of events	<4	6	<4
Other/unknown			
No. of patients (%)	35 (62.5)	40 (39.6)	27 (30.3)
No. of events	44	49	27

Reasons were available for 163/169 patients in the all-comers cohort. More than 1 episode and/or more than 1 reason could have been recorded per patient, so the percentages sum to >100%.

Abbreviations: No., number.

A total of 36 patients (of 169 [21.3%]) had records of CYP3A4 inhibitor administration in the ± 30-day window around AML diagnosis. Of 72 patients with venetoclax dosing <400 mg, 23 (32%) had concomitant treatment with CYP3A4 inhibitors documented in the EHR. Of the 15 patients with strong CYP3A4 inhibitor use (eg, voriconazole or posaconazole), 11 (73.3%) received venetoclax doses of ≤100 mg, and of the 14 patients with moderate CYP3A4 inhibitor use (eg, fluconazole or isavuconazonium sulfate), 13 (92.9%) received venetoclax at a dose of 200 mg. A full list of CYP3A4 inhibitors searched for is available in Supplementary Table S2.

### Treatment Duration

**Figure 3.**
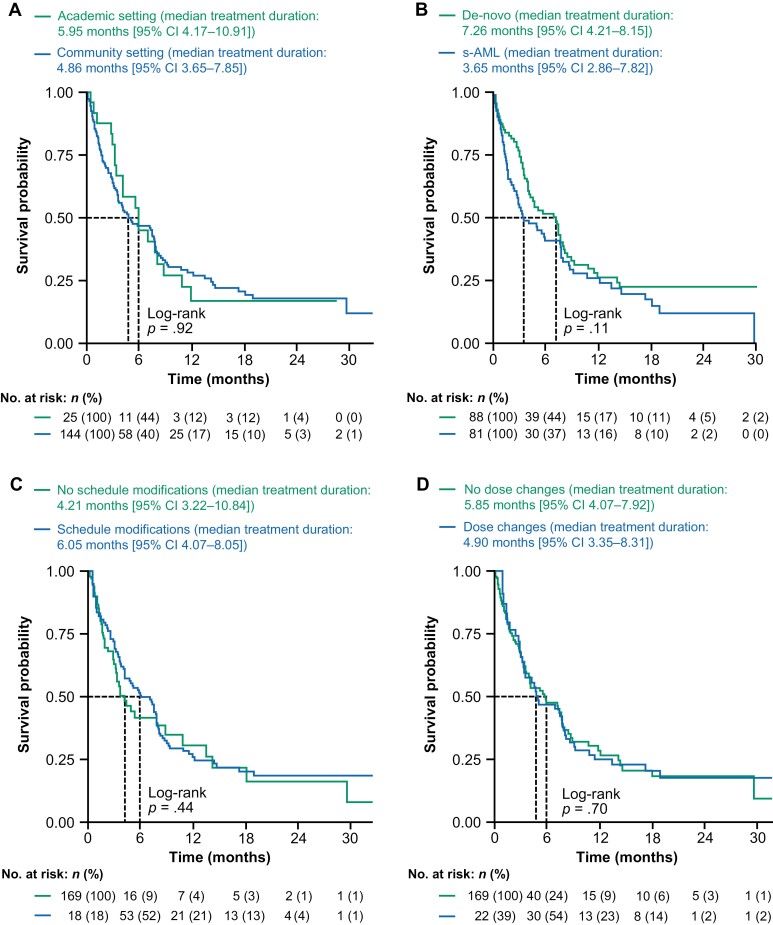
Median treatment duration according to practice type **(A)**, s-AML versus de-novo AML **(B)**, presence or absence of venetoclax dose schedule modifications **(C)**, and the presence or absence of venetoclax dose changes **(D)**. Abbreviations: AML, acute myeloid leukemia; CI, confidence interval; no., number; s-AML, secondary acute myeloid leukemia.

With a median follow-up of 7.2 months, the median treatment duration was 5.2 months (95% CI = 4.0-7.7) for patients treated with venetoclax in the all-comers cohort (Supplementary Fig. S2A). There were no significant differences between groups in unadjusted analyses stratified by baseline characteristics, including age (<75 years versus ≥75 years; Supplementary Fig. S2B), prior use of HMAs for patients with s-AML (prior HMA use versus no prior HMA; Supplementary Fig. S2C), practice type (Fig. 3A), and AML type (s-AML or de-novo; Fig. 3B). In time-varying analyses, there was no significant difference in median treatment duration between the 101 patients with treatment schedule modifications (6.1 months [95% CI = 4.1-8.1]) and those who did not modify their treatment schedule (4.2 months [95% CI = 3.2-10.8]; Fig. 3C), or between the 56 patients with dose changes (4.9 months [95% CI = 3.4-8.3]) and those without dose changes (*n* = 113, 5.9 months [95% CI = 4.1-7.9]; Fig. 3D).

### Response to Therapy

Of the 95 patients with BM blast-evaluable data during follow-up, 51 (53.7%) had their first biopsy by day 28 (±14) of treatment (a proxy for BM around the first treatment cycle), the majority of whom (41/51; 80.4%) achieved a BM response (blast clearance) at that time. Delay in BM assessment was associated with a lower change in blast count from baseline (29/34 [85.3%] of patients with BM evaluation within 30 days of diagnosis were responders, 22/36 [61.1%] with BM assessment within 30-60 days were responders, and 10/20 [50.0%] patients with BM assessment within 60-90 days were responders). Most of the BM biopsy evaluations that occurred after 90 days were repeat/follow-up biopsies.

Seventy-eight of the 95 (82.1%) patients with BM blast-evaluable data available at any time during follow-up had a BM response; the remaining patients (17/95; 17.9%) did not have BM responses (Fig. 2). Of the 78 patients who had BM responses, 45 (57.7%) had a rwCR/CRh (Fig. 2). Treatment modifications occurred for 25 (55.6%) patients with rwCR/CRh within the 30 days following remission: 7 patients had dose holds/interruptions, 8 had cycle delays, 6 had schedule changes, and the remaining 4 had dose changes or discontinued treatment.

### Survival

**Figure 4.**
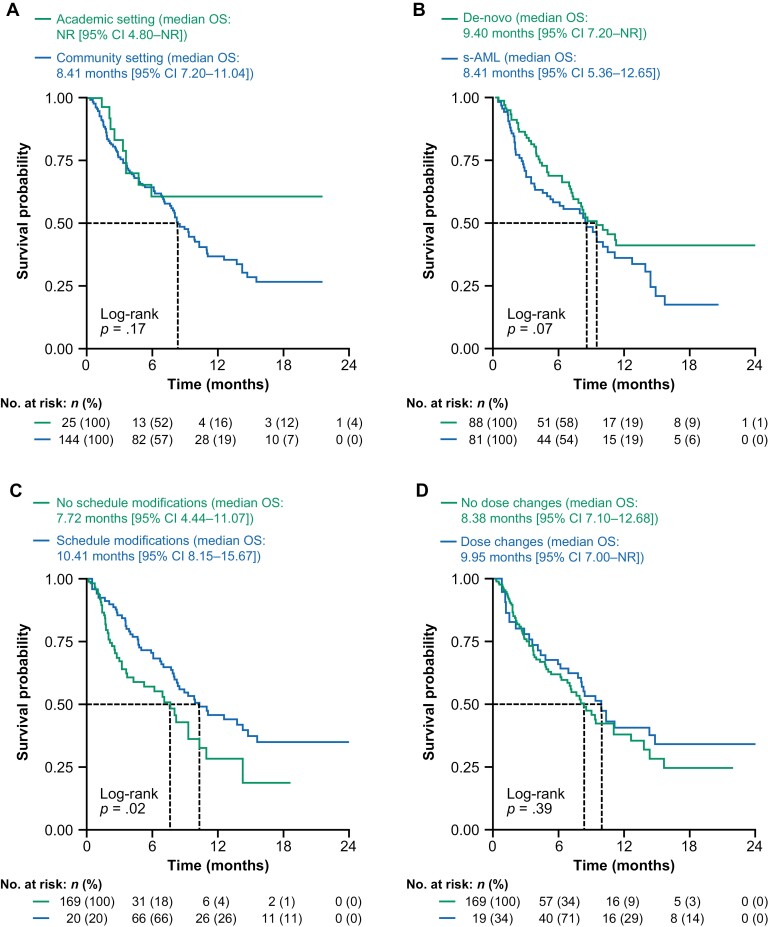
Median OS according to practice type **(A)**, s-AML versus de-novo AML **(B)**, presence or absence of venetoclax treatment schedule modifications **(C)**, and presence or absence of venetoclax dose changes **(D)**. Abbreviations: AML, acute myeloid leukemia; CI, confidence interval; no., number; NR, not reached; OS, overall survival; s-AML, secondary acute myeloid leukemia.

The observed median OS was 8.6 months (95% CI = 7.7-11.1) for patients treated with venetoclax in the all-comers cohort (Supplementary Fig. S3A). There were no significant differences in key subgroups based on baseline characteristics, including age (<75 years versus ≥75 years; Supplementary Fig. S3B), practice type (Fig. 4A), and AML type (s-AML or de-novo; Fig. 4B). Among the 75 patients with s-AML, however, those with prior HMA use (*n* = 20) had a significantly shorter median OS (3.8 months [95% CI = 1.8-10.0]) versus those who had no record of prior HMAs (*n* = 55; 12.6 months [95% CI = 5.4-not reached (NR)]; log-rank *P = .*0018; Supplementary Fig. S3C).

Time-varying covariate analyses, showed that patients with treatment schedule modifications (*n* = 101) had a significantly longer median OS (10.4 months [95% CI = 8.2-15.7]) compared with those who did not modify their treatment schedule (7.7 months [95% CI = 4.4-11.1]; *P* = .02; Fig. 4C). Although the 56 patients with dose changes had a numerically longer median OS (10.0 months [95% CI = 7.0-NR]) versus those without dose changes (8.4 months [95% CI = 7.1-12.7]; Fig. 4D), there was no significant difference in survival.

### Cytopenias

A subset of 48 patients had complete laboratory data available at baseline and in follow-up and thus could be examined for the occurrence of treatment-emergent grade 3-4 cytopenias. A total of 44 patients had a treatment-emergent event, at a median of 8 days (95% CI = 6-15) after the start of therapy, with most (39 [88.6%] patients) occurring within 60 days of treatment initiation.

## Discussion

This study reports on the early real-world experience of 169 patients with newly diagnosed AML who were treated with venetoclax + HMAs prior to data availability from the pivotal phase III VIALE-A trial. This analysis therefore reflects early experience and management with the combination therapy, predominantly in the US community setting.

The baseline characteristics of this patient cohort were as expected in terms of age, cytogenetic characteristics, and ECOG PS. Compared with clinical trial populations, this predominately community-based cohort was older and included more cases of s-AML,^11,13^ including those previously treated with HMA monotherapy. In this real-world population, a large proportion of patients (43.8%) were unevaluable for blast clearance during the follow-up period (due to a lack of BM data in their records, which is an inherent limitation of real-world data collection). In the evaluable patients with BM assessment during follow-up (BM blast-evaluable group), a relatively high response rate was observed, and around half of the patients had a recorded BM result after the first cycle of treatment. Twelve (of 169 [7.1%]) patients bridged to transplant, which was higher than observed in the VIALE-A clinical trial (where 2/283 [0.7%] patients in the venetoclax + azacitidine arm and 1/144 [0.7%] in the placebo + azacitidine arm underwent transplantation after discontinuing treatment)^11^; however, the eligibility criteria of VIALE-A excluded transplant-eligible patients. Considering the curative potential of transplant, it is encouraging to observe that some patients could proceed to transplant after treatment with venetoclax + HMA, even in this real-world cohort of older patients treated primarily in the community setting.

In the current study, most patients had changes to venetoclax dosing and/or treatment schedules over the course of therapy. The median real-world treatment duration on venetoclax (5.2 months), follow-up time (7.2 months) and median OS (8.6 months) were shorter than in the phase III VIALE-A trial (7.0 cycles median treatment duration, 20.5 months median follow-up, and 14.7 months median OS),^11^ likely due to the differences in patient populations, shorter follow-up time, and limitations to this real-world study as described. Although venetoclax treatment modifications did not affect treatment duration in the current study, patients with treatment schedule modifications had a significantly longer median OS compared with those without, and patients with venetoclax dose changes had a numerically (non-significantly) longer median OS versus those without. This reflects the importance of treatment management to mitigate toxicity.

Notably, there are 2 important differences between the VIALE-A population and the patients in the current study: 25% of patients in VIALE-A had s-AML compared with 43.8% in this cohort (most of whom [59/74; 79.7%] had myelodysplastic syndrome), and VIALE-A did not include patients who had previous HMA therapy.^11^ While median OS was numerically longer for de-novo AML versus s-AML in this study, median OS was numerically shorter for de-novo AML versus s-AML in VIALE-A; there was no statistical difference in either study. The s-AML population with previous HMA therapy reported here had very poor outcomes (median OS <4 months). Previous analyses we have conducted showed a poorer median OS for patients who have received 1L HMA (6.3 months) in the Flatiron Health AML database compared with clinical trials of HMA.^27^ The heterogeneous, less fit population in the current study (with a greater proportion of patients with s-AML, including those previously treated with HMAs, who are known to have poorer outcomes), could explain the short real-world duration of therapy versus the VIALE-A trial.^27^ Due to the selective eligibility criteria and tightly controlled settings for clinical trials,^28^ better management and superior outcomes compared with real-world studies are to be expected.

In contrast, another real-world study, the AML Real-world Evidence (ARC) Initiative study (which examined patients with newly diagnosed AML in a real-world academic setting), reported treatment outcomes similar to those observed in VIALE-A (overall response rate [CR + CRh + CRi] was 58.5% in patients in the ARC study receiving low-intensity regimens with venetoclax versus 64.7% of patients who achieved a CR + CRh in VIALE-A).^29^ Efficient and comprehensive access to diagnostics and therapies, including transplant, as well as the availability of resources for supportive care and management by multidisciplinary teams with disease expertise, has been suggested to lead to improved outcomes at academic centers.^30^ Therefore, it is not surprising that outcomes for the ARC initiative were more aligned with clinical trials than with the outcomes for this current study (in which the large majority of the patients were treated in the community setting). This difference in outcomes within the real-world studies also highlights the importance of optimizing management with venetoclax, regardless of patient setting.

In the current study, around half of patients started on the recommended dose of 400 mg venetoclax, suggesting that azole prophylaxis was deferred or not received by many patients. However, not all patients on lower doses had documented CYP3A4 inhibitor treatment in their EHR. It is possible that these prescriptions were filled outside the oncology practice for patients on lower doses of venetoclax, or indeed those receiving 400 mg venetoclax, and hence may not have been captured in the oncology EHR, but could have resulted in cytopenias with increased venetoclax exposure. The recorded reasons for dose/treatment schedule changes were predominantly due to the toxic effects of therapy. Drug–drug interactions were also a reason for venetoclax dose modification, particularly dose changes, and we observed correct modifications of venetoclax dosing according to the use of strong or moderate CYP3A4 inhibitors, where data were evaluable. Treatment schedule modifications were common early in the treatment course and were associated with better outcomes in time-varying analyses.

There are some limitations to this study. An EHR-derived database was used; hence data are not captured specifically for research purposes. The data abstracted were as recorded in the oncology medical records, and lack of documentation of treatment or procedures does not mean that these did not occur; eg, BM assessments and CYP3A4 inhibitor use. In addition, important clinical data, such as minimal residual disease measurements, and real-world quality of life outcomes, and total hospital burden (relevant from the patient perspective),^31^ were not captured in the database, so they were unavailable for analysis. The Flatiron Health database is an open cohort and as such, some patients may have visited other sites of care. Information about patients’ treatment outside of the Flatiron Health network may not be captured in the data and therefore may lead to underreporting or missing data, which may explain some of the undocumented BM assessments in follow-up. This is also a reason for not having structured laboratory data for all patients throughout their course of care, so the assessment of treatment-emergent cytopenias is in a limited cohort and subject to informed presence bias.

## Conclusions

This study represents the early experience of venetoclax treatment in a US setting with patients predominantly attending community oncology centers. Most venetoclax-treated patients had dose changes or modifications to their venetoclax treatment regimen, mainly due to toxicities or drug–drug interactions. Only half of the patients with documented BM biopsy were assessed at approximately cycle 1, but a high response rate was observed in evaluated patients. While the real-world cohort reported here had a shorter follow-up time and median OS than reported in clinical trials, patients with treatment schedule modifications had longer OS versus those without. These observations highlight the importance of appropriate venetoclax management (including early BM assessment and dose schedule modifications for toxicities) to optimize patient outcomes.

## Supplementary Material

oyac135_suppl_Supplementary_Material

## Data Availability

The de-identified data that support the findings of this study are subject to a license agreement with Flatiron Health; interested researchers should contact DataAccess@flatiron.com to determine licensing terms.

## References

[1] Juliusson G , Antunovic P, Derolf A, et al. Age and acute myeloid leukemia: real world data on decision to treat and outcomes from the Swedish Acute Leukemia Registry. Blood. 2009;113(18):4179-4187. 10.1182/blood-2008-07-172007. 19008455

[2] Juliusson G , Lazarevic V, Hörstedt AS, et al. Acute myeloid leukemia in the real world: why population-based registries are needed. Blood. 2012;119(17):3890-3899. 22383796 10.1182/blood-2011-12-379008 PMC3358248

[3] Board CNE. Leukemia—Acute Myeloid—AML: Statistics. Available at https://www.cancer.net/cancer-types/leukemia-acute-myeloid-aml/statistics. Accessed January 2022.

[4] Döhner H , Estey E, Grimwade D, et al. Diagnosis and management of AML in adults: 2017 ELN recommendations from an international expert panel. Blood. 2017;129(4):424-447. 27895058 10.1182/blood-2016-08-733196 PMC5291965

[5] Fröhling S , Schlenk RF, Kayser S, et al. Cytogenetics and age are major determinants of outcome in intensively treated acute myeloid leukemia patients older than 60 years: results from AMLSG trial AML HD98-B. Blood. 2006;108(10):3280-3288. 16840728 10.1182/blood-2006-04-014324

[6] Thein MS , Ershler WB, Jemal A, et al. Outcome of older patients with acute myeloid leukemia: an analysis of SEER data over 3 decades. Cancer. 2013;119(15):2720-2727. 23633441 10.1002/cncr.28129 PMC3821042

[7] Lang K , Earle CC, Foster T, et al. Trends in the treatment of acute myeloid leukaemia in the elderly. Drugs Aging. 2005;22(11):943-955. 16323971 10.2165/00002512-200522110-00004

[8] US Food and Drug Administration. FDA Approves Venetoclax in Combination for AML in Adults. 2018. Available at https://www.fda.gov/drugs/fda-approves-venetoclax-combination-aml-adults. Accessed January 2022.

[9] AbbVie. AbbVie Receives US FDA Accelerated Approval for VENCLEXTA® (venetoclax) for Treatment of Newly-Diagnosed Acute Myeloid Leukemia Patients Ineligible for Intensive Chemotherapy. 2018. Available at https://news.abbvie.com/news/press-releases/abbvie-receives-us-fda-accelerated-approval-for-venclexta-venetoclax-for-treatment-newly-diagnosed-acute-myeloid-leukemia-patients-ineligible-for-intensive-chemotherapy.htm. Accessed January 2022.

[10] AbbVie. VENCLEXTA® (venetoclax) Receives FDA Full Approval for Acute Myeloid Leukemia (AML) 2020. Available at https://news.abbvie.com/news/press-releases/venclexta-venetoclax-receives-fda-full-approval-for-acute-myeloid-leukemia-aml.htm. Accessed January 2022.

[11] DiNardo CD , Jonas BA, Pullarkat V, et al. Azacitidine and venetoclax in previously untreated acute myeloid leukemia. N Engl J Med. 2020;383(7):617-629. 32786187 10.1056/NEJMoa2012971

[12] Pollyea DA , Bixby D, Perl A, et al. NCCN guidelines insights: Acute myeloid leukemia, version 2.2021. J Natl Compr Canc Netw. 2021;19(1):16-27. 33406488 10.6004/jnccn.2021.0002

[13] DiNardo CD , Pratz K, Pullarkat V, et al. Venetoclax combined with decitabine or azacitidine in treatment-naive, elderly patients with acute myeloid leukemia. Blood. 2019;133(1):7-17. 30361262 10.1182/blood-2018-08-868752 PMC6318429

[14] Rauch PJ , Ellegast JM, Widmer CC, et al. MPL expression on AML blasts predicts peripheral blood neutropenia and thrombocytopenia. Blood. 2016;128(18):2253-2257. 27574191 10.1182/blood-2016-04-711986

[15] Goldberg AD , Tallman MS. Down for the count in acute myeloid leukemia. Blood. 2016;128(18):2195-2197. 27811189 10.1182/blood-2016-09-738765

[16] Rausch CR , DiNardo CD, Maiti A, et al. Duration of cytopenias with concomitant venetoclax and azole antifungals in acute myeloid leukemia. Cancer. 2021;127(14):2489-2499. 33793970 10.1002/cncr.33508 PMC8249340

[17] Dombret H , Seymour JF, Butrym A, et al. International phase 3 study of azacitidine vs conventional care regimens in older patients with newly diagnosed AML with >30% blasts. Blood. 2015;126(3):291-299. 25987659 10.1182/blood-2015-01-621664 PMC4504945

[18] Kantarjian HM , Thomas XG, Dmoszynska A, et al. Multicenter, randomized, open-label, phase III trial of decitabine versus patient choice, with physician advice, of either supportive care or low-dose cytarabine for the treatment of older patients with newly diagnosed acute myeloid leukemia. J Clin Oncol. 2012;30(21):2670-2677. 22689805 10.1200/JCO.2011.38.9429 PMC4874148

[19] Fenaux P , Mufti GJ, Hellstrom-Lindberg E, et al. Efficacy of azacitidine compared with that of conventional care regimens in the treatment of higher-risk myelodysplastic syndromes: a randomised, open-label, phase III study. Lancet Oncol. 2009;10(3):223-232. 19230772 10.1016/S1470-2045(09)70003-8 PMC4086808

[20] Kantarjian H , Issa JP, Rosenfeld CS, et al. Decitabine improves patient outcomes in myelodysplastic syndromes: results of a phase III randomized study. Cancer. 2006;106(8):1794-1803. 16532500 10.1002/cncr.21792

[21] Pratz KW , DiNardo CD, Selleslag D, et al. Cytopenia management in patients with newly diagnosed acute myeloid leukemia treated with venetoclax plus azacitidine in the VIALE-A study. Blood. 2020;136(Suppl. 1):51-53.

[22] Juárez-Salcedo LM , Desai V, Dalia S. Venetoclax: evidence to date and clinical potential. Drugs Context. 2019;8:212574. 31645879 10.7573/dic.212574 PMC6788387

[23] Richard-Carpentier G , DiNardo CD. Venetoclax for the treatment of newly diagnosed acute myeloid leukemia in patients who are ineligible for intensive chemotherapy. Ther Adv Hematol. 2019;10:2040620719882822. 31692757 10.1177/2040620719882822 PMC6811760

[24] Agarwal SK , DiNardo CD, Potluri J, et al. Management of venetoclax-posaconazole interaction in acute myeloid leukemia patients: evaluation of dose adjustments. Clin Ther. 2017;39(2):359-367. 28161120 10.1016/j.clinthera.2017.01.003

[25] Birnbaum B , Nussbaum N, Seidl-Rathkopf K, et al. Model-assisted cohort selection with bias analysis for generating large-scale cohorts from the EHR for oncology research. arXiv; 2020;arXiv:2001.09765. 10.48550/arXiv.2001.09765

[26] Ma X , Long L, Moon S, et al. Comparison of population characteristics in real-world clinical oncology databases in the US: Flatiron Health, SEER, and NPCR. medRxiv; 2020;03.16.20037143. 10.1101/2020.03.16.20037143.

[27] Pardee TS , Oschwald J, Ma E, et al. Treatment patterns and outcomes of patients with newly diagnosed acute myeloid leukemia (AML) treated with hypomethylating agents (HMA) in the United States (US). Blood 2020;136(Suppl. 1):14-16.

[28] Lee J , Park BA. method to evaluate scientific evidence in clinical preventive medicine. JKMA. 2011;54(10):1006-1012.

[29] Garcia JS , Wolach O, Vachhani P, et al. Poster 2328 Comparative effectiveness of venetoclax combinations vs other therapies among patients with newly diagnosed acute myeloid leukemia: Results from the AML Real World Evidence (ARC) Initiative. Presented at American Society of Hematology (ASH) Annual Meeting & Exposition, 2021.

[30] Halpern AB , Walter RB. Practice patterns and outcomes for adults with acute myeloid leukemia receiving care in community vs academic settings. Hematology Am Soc Hematol Educ Program. 2020;2020(1):129-134. 33275691 10.1182/hematology.2020000097 PMC7727552

[31] Wei AH , Roboz GJ, Kantarjian HM. Harnessing the therapeutic value of venetoclax: a breakthrough therapy in acute myeloid leukemia. J Clin Oncol. 2021;39(25):2742-2748. 34086506 10.1200/JCO.21.00080 PMC9851684

