# Supplementary material for: Venetoclax and Hypomethylating Agents as First-line Treatment in Newly Diagnosed Patients with AML in a Predominately Community Setting in the US
Source: Oncologist. 2022 Aug 4;27(11):907–18. doi: 10.1093/oncolo/oyac135 (PMC9632323; doi:10.1093/oncolo/oyac135)
Supplement: oyac135_suppl_Supplementary_Material [file oyac135_suppl_supplementary_material.docx]

**SUPPLEMENTAL DATA**

**Supplementary Methods**

***AML Diagnosis***

Diagnosis of AML during clinical visits was established in line with the International Classification of Disease (ICD) version 9, codes 205.0x, 205.9x, 206.0x, 207.0x and 207.2x, and ICD version 10 codes C92.0x, C92.4x, C92.5x, C92.6x, C92.9x, C92.Ax, C93.0x, C94.0x, C94.2x and C94.4x.

**Supplementary Tables**

**Supplementary Table S1.** Variables included in the data model

| **Source** | **Variable** |
| --- | --- |
| Unstructured data from EHR related to venetoclax treatment | - Dosing information - Duration of therapy^a^ - Occurrence and timing of dose changes (in mg) and schedule changes - Reasons for dose holds (interruptions), dose and schedule changes - Occurrence and timing of discontinuation and reasons for discontinuation |
|  | - Dose schedule modification - In-cycle dose interruptions: dose hold in the middle of a cycle - Drug holidays: dose hold within the last days of a cycle – where the next cycle starts no more than 31 days after the start of the previous cycle - Dose cycle conversion: where number of dosing days per cycle changes from one cycle to another, e.g., a 28-day cycle converts to a 21-day cycle - Cycle delay: dose hold within the last days of a cycle that delays the start of the next cycle |
|  | - Drug–drug interactions,^b^ e.g., the co-administration of CYP3A4 inhibitors with venetoclax |
| Other information | - HMA dosing information |
|  | - Cytopenia determined from structured laboratory data in the EHR where available - Cytopenia events were grade 3 or 4 neutropenia (absolute neutrophil count of ≥0.5–<1.0 x 10^9^/L or <0.5 x 10^9^/L, respectively) and/or grade 3 or 4 thrombocytopenia (platelet count of ≥25.0–<50.0 or <25.0 x 10^9^/L, respectively) |
|  | - Timing of BM biopsy/assessment - Response to treatment was measured as “blast clearance” ≤5% from BM biopsy after the index date |
|  | - rwCR/CRh - Derived as ≤5% BM blasts with at least partial hematologic count recovery (platelet count of >50 × 10^9^/L and absolute neutrophil count of >0.5 × 10^9^/L) within 14 days of biopsy |
|  | - Treatment duration - Time from treatment initiation to last administration before an absence of treatment, discontinuation, or death |
|  | - OS - Time from treatment initiation to death date where available or censoring at last activity date (treatment or visit) before end of follow-up |

^a^From start date until discontinuation or end of follow-up, including summary of dosing cycles (based on the first HMA administration and on a cadence of 28 ± 3 days) and dosing interruptions (number of days without exposure to venetoclax).

^b^Where drug–drug interactions were cited as a reason for dose modification, the specific CYP3A4 inhibitor therapy was abstracted where recorded.

Abbreviations: BM, bone marrow; EHR, electronic health record; HMA, hypomethylating agent; OS, overall survival; rwCR/CRh, real-world complete response/complete response with partial hematologic recovery.

**Supplementary Table S2**. CYP3A4 inhibitors used

| **Strength of inhibitor** | **Name** |
| --- | --- |
| Strong | Itraconazole |
|  | Ketoconazole |
|  | Posaconazole |
|  | Voriconazole |
|  | Clarithromycin |
| Moderate | Ciprofloxacin |
|  | Fluconazole |
|  | Erythromycin |
|  | Isavuconazonium sulfate |
|  | Diltiazem |
|  | Verapamil |

**Supplementary Figure S1**. Cumulative percentage of patients with treatment schedule modification from 28 days to any other (shorter) treatment schedule.


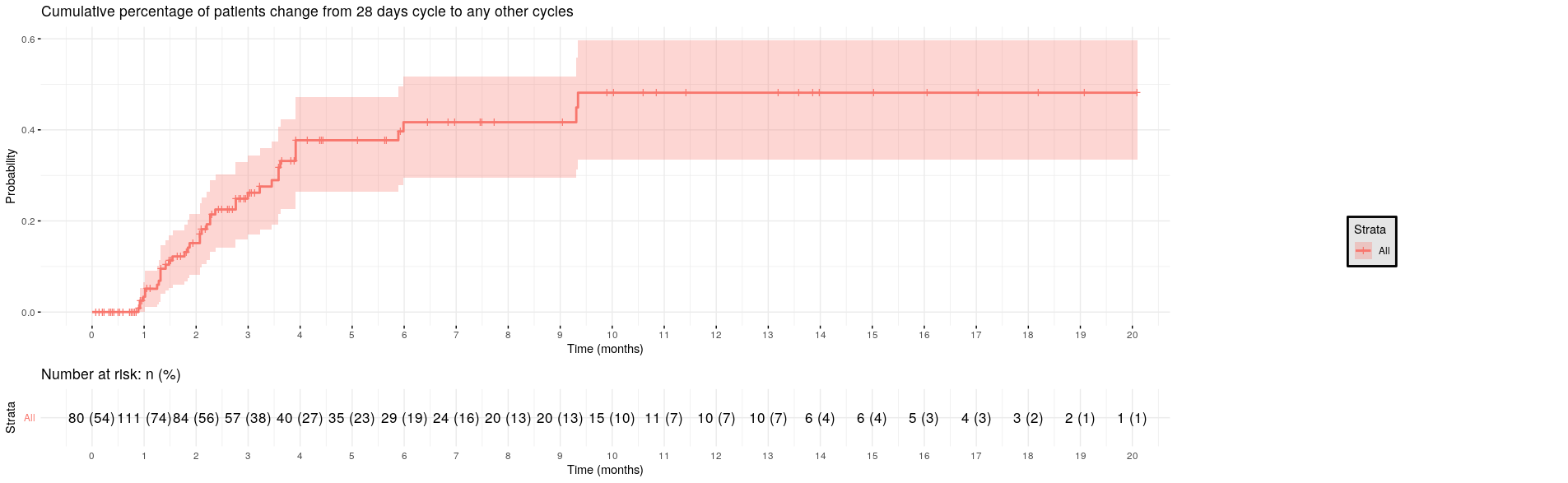


| ***N*** | **Events** | **Median** | **Lower95** | **Upper95** |
| --- | --- | --- | --- | --- |
| 149 | 39 | NA | 182 | NA |

Ticks mark censoring at treatment discontinuation.
Abbreviations: NA, not applicable.

**Supplementary Figure S2**. Median treatment duration among the all-comers cohort **(A)**, in patients aged <75 versus ≥75 years **(B)**, and in patients with s-AML with versus without prior HMA treatment **(C)**.

**A**

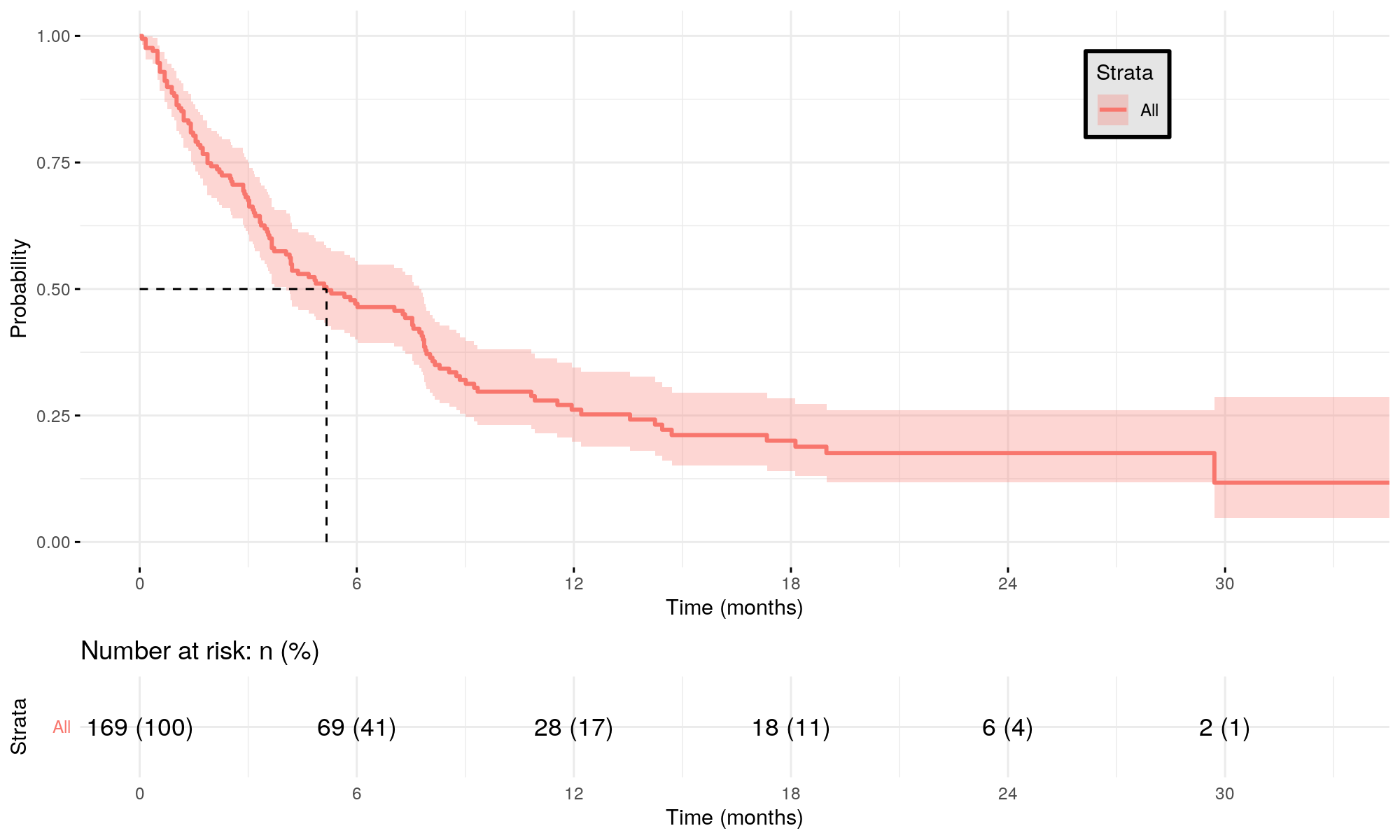


**B**


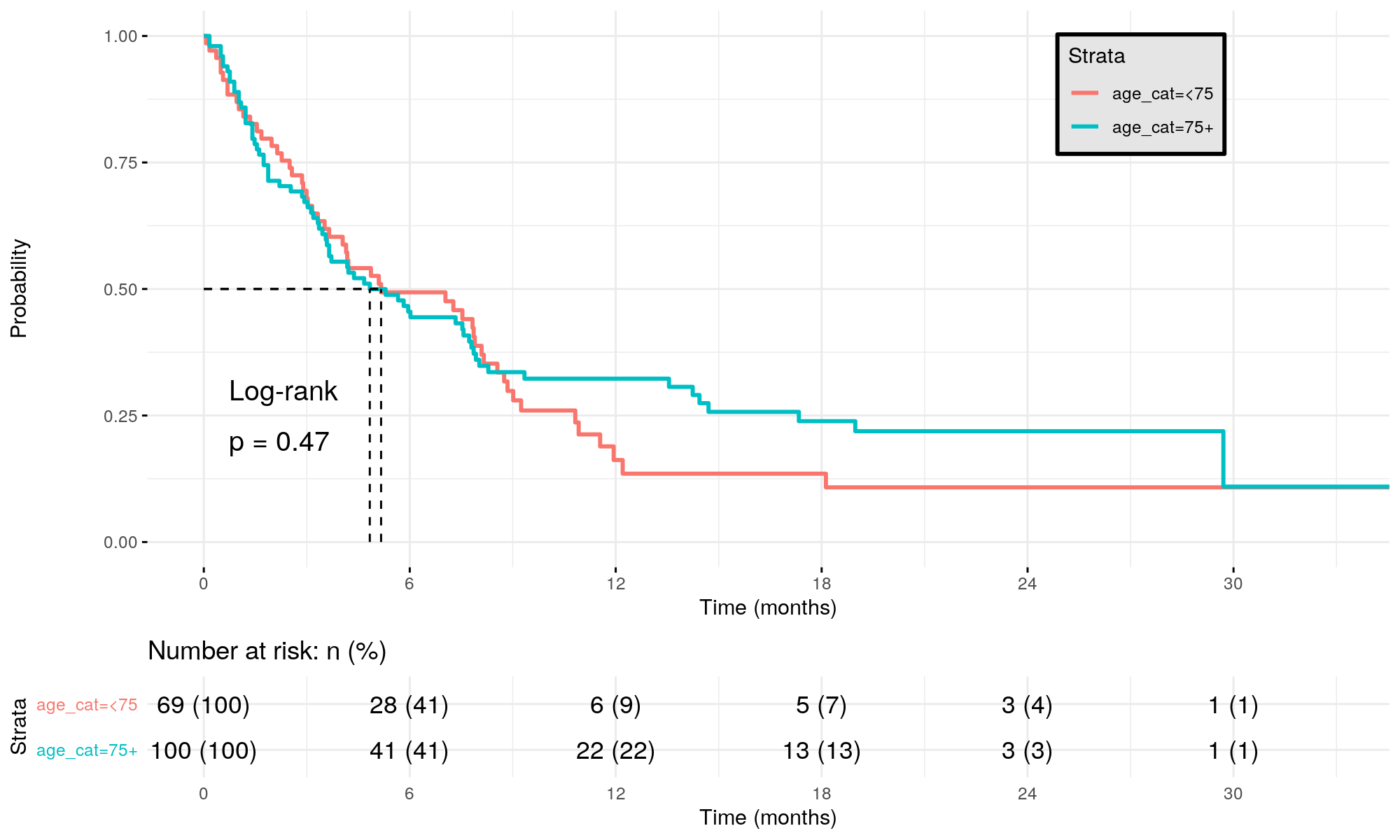


**C**

​​
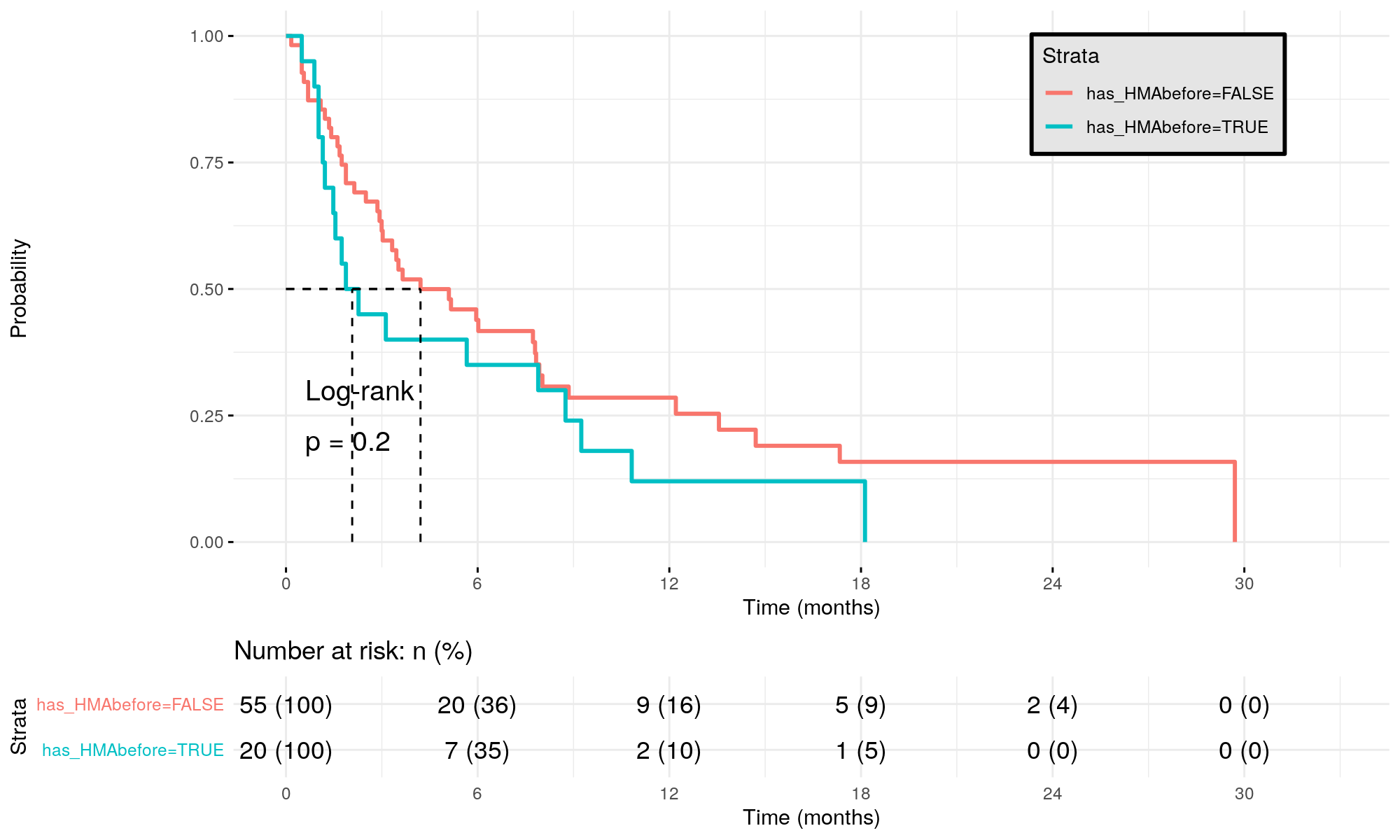


Abbreviations: HMA, hypomethylating agent; s-AML, secondary acute myeloid leukemia.

**Supplementary Figure S3**. Median OS among the all-comers cohort **(A)**, in patients aged <75 versus ≥75 years **(B)**, and in patients with s-AML with versus without prior HMA treatment **(C)**.

**A**

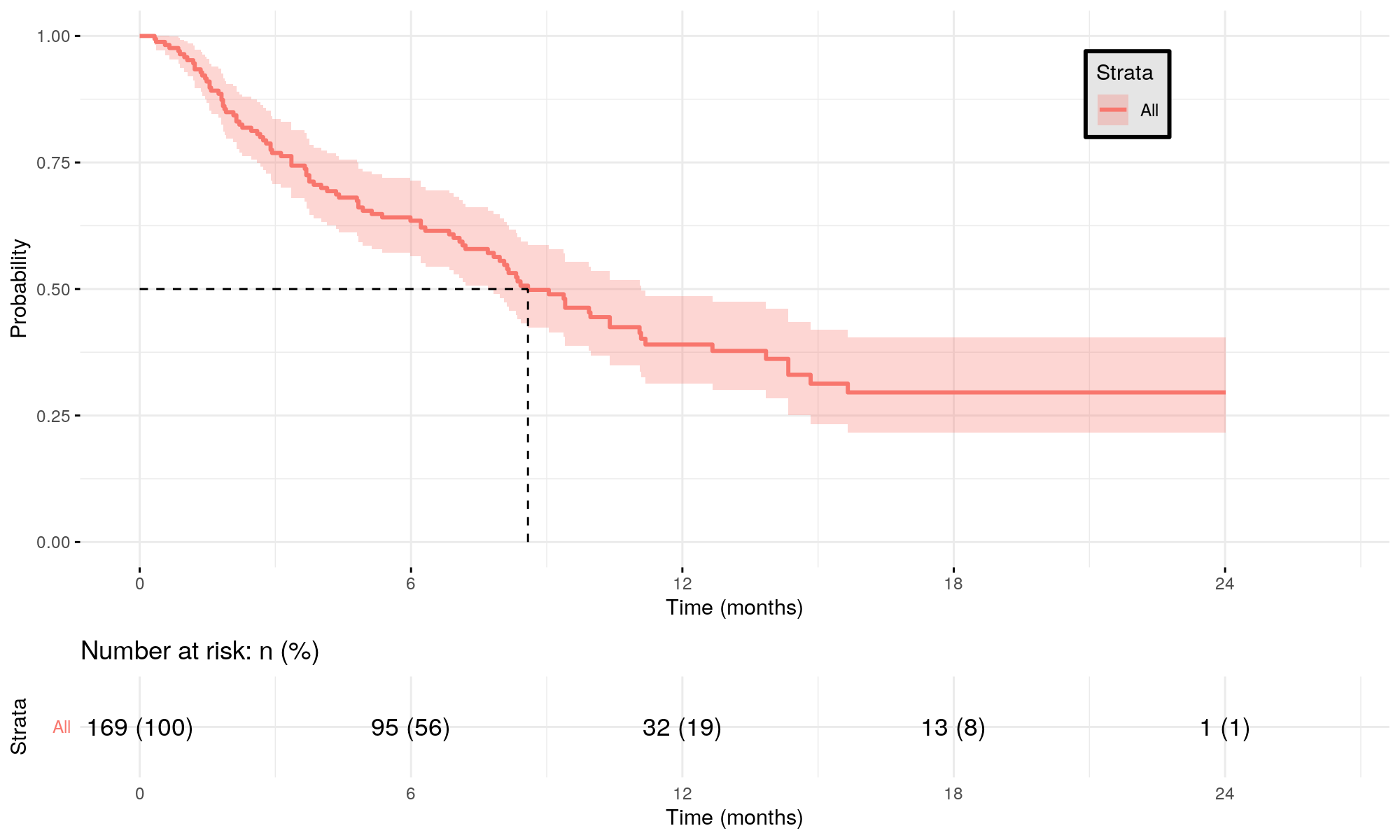


**B**
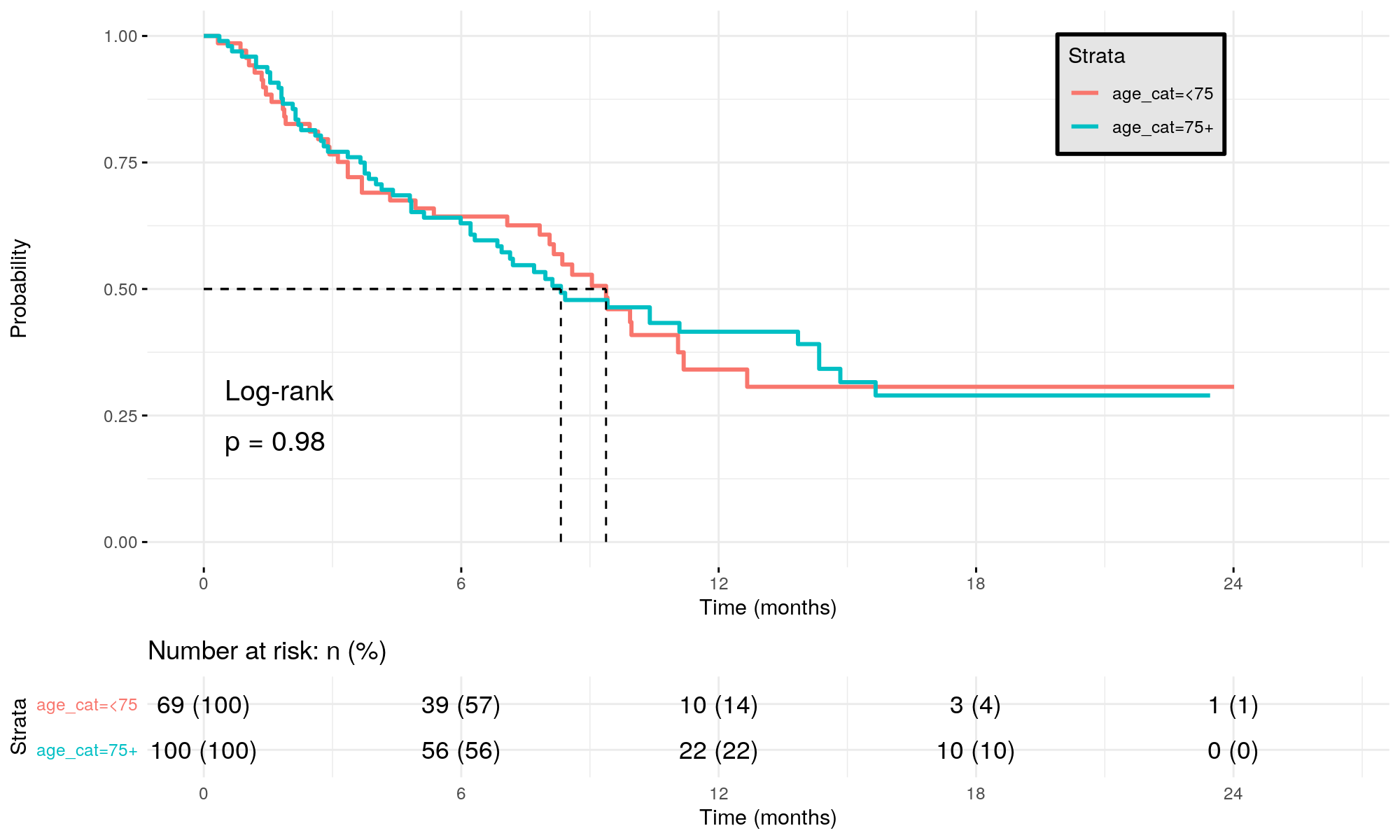


**C**


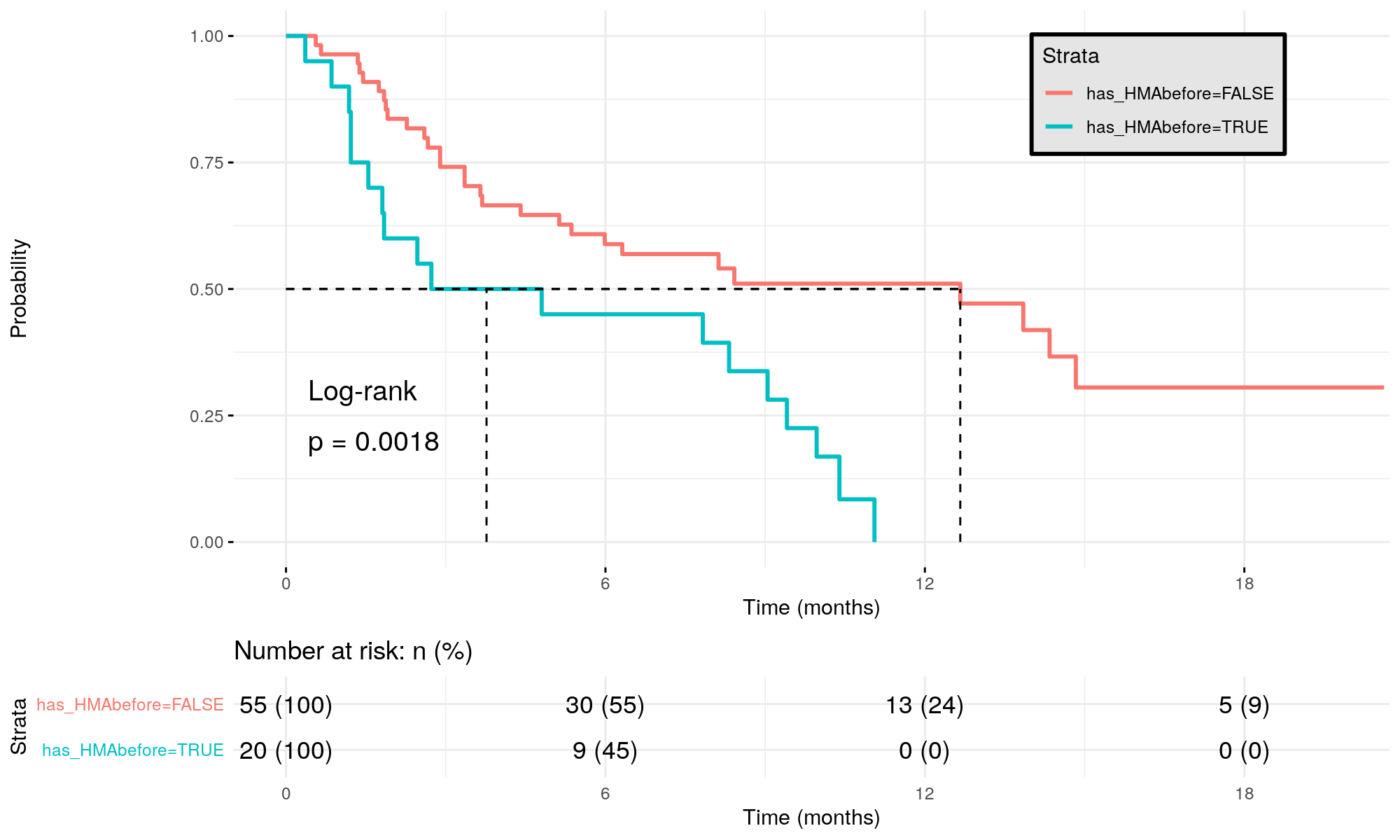


Abbreviations: HMA, hypomethylating agent; OS, overall survival; s-AML, secondary acute myeloid leukemia.
